# Seasonal variability in essential oil composition and biological activity of *Rosmarinus officinalis* L. accessions in the western Himalaya

**DOI:** 10.1038/s41598-022-07298-x

**Published:** 2022-02-28

**Authors:** Shalika Rathore, Srijana Mukhia, Smita Kapoor, Vinod Bhatt, Rakshak Kumar, Rakesh Kumar

**Affiliations:** 1 Agrotechnology Division, CSIR-Institute of Himalayan Bioresource Technology, Palampur, Himachal Pradesh 176061 India; 2 Biotechnology Division, Department of Microbiology, Guru Nanak Dev University, Amritsar, 143 005 Punjab India; 3 Dietetics and Nutrition Technology, CSIR-Institute of Himalayan Bioresource Technology, Palampur, Himachal Pradesh 176061 India; 4 Chemical Technology Division, CSIR-Institute of Himalayan Bioresource Technology, Council of Scientific and Industrial Research, Palampur, 176 061 Himachal Pradesh India; 5 Biotechnology Division, CSIR-Institute of Himalayan Bioresource Technology, Palampur, Himachal Pradesh 176061 India; 6 Academy of Scientific and Innovative Research, Ghaziabad, Uttar Pradesh 201002 India

**Keywords:** Biological techniques, Plant sciences

## Abstract

*Rosmarinus officinalis* L. is an imperative herb used in pharmaceutical yet knowledge on chemical and activity profile of essential oil (EO) to harvest seasons and accessions from the Himalayan region is limited. Thus, accessions were evaluated to determine the EO content, compositional, antimicrobial, and cytotoxic potential of rosemary in different harvest seasons during 2018‒2019. EO content was 30.5% higher in IHBT/RMAc-1 compared with IHBT/RMAc-2 accession while 27.9% and 41.6% higher in the autumn as compared with summer and rainy season, respectively. Major EO compound was 1,8-cineole; ranged from 32.50‒51.79% during harvest seasons and 38.70‒42.20% in accessions. EO was active against both the tested Gram-positive bacteria (*Micrococcus* *luteus* MTCC 2470 and *Staphylococcus aureus* MTCC 96). EOs showed inhibition of Gram-negative bacteria (*Salmonella typhi* MTCC 733), while *Klebsiella pneumoniae* MTCC 109 was found to be resistant. The rosemary EO of T1 (Rainy season IHBT/RMAc-1) was most effective against *S. aureus* MTCC 96 with the minimum inhibitory concentration (MIC) of 4% (v/v). In vitro cytotoxicity evaluation showed no potential anti-proliferative activity of EO. The rosemary EO profile in the western Himalayan region was influenced by harvesting seasons and genetic variability within the accessions; furthermore, a promising antibacterial agent in pharmaceutical and flavour industries.

## Introduction

Aromatic and medicinal plants are the repository of numerous secondary metabolites valuable for mankind. Essential oils (EOs) from these plants have great importance in several areas, viz., natural food-flavour, preservatives, perfumery, aromatherapy, pharmaceuticals, and related medicinal uses^1^. There is a huge demand for EO in the global market, increasing at a 9.3% compound annual growth rate; furthermore, projected to reach USD 16.0 billion by 2026^2^. One such perennial herbaceous plant with huge potential is rosemary (*Rosmarinus officinalis* L.) (Family: Lamiaceae). Aerial parts of *R. officinalis* contain EO and phenolic compounds which possesses^3^, antimicrobial, and antitumor properties^4^. The key EO constituents are 1,8-cineole, camphor, borneol, β-caryophyllene, and composition of the EO is known to fluctuate with the season^5^, climate, land, soil^6^, and developmental stages^7^. The influence of harvest time and seasons on EO composition on a few aromatic crops viz., Turkish oregano^8^, thyme^9^, and *Ocimum* spp.^10^ were broadly researched. Moreover, Himalayan regions are an important factor in the improvement of EO quality and productivity, and therefore, EO finds different uses depending on specific components’ availability^11,12^, and the demand for unique and distinctive EO producing cultivars has also increased^13^. Earlier studies reported variations in EO composition among rosemary populations were based on genetic history, varietal difference^14^, ecological/environmental disparity^15^. The seasonal variability in various locations of Serbia^16^ and Italy^5^ were also studied in rosemary. In the present study, the seasonal variability in the Himalayan region may also be possible to affirm that rosemary accessions in this distinct region could have a differentiated EO profile. Furthermore, the EO distilled from *Tagetes minuta* L. grown at lower altitude regions was reported to have dihydrotagetone followed by tagetone and Z-β-ocimene^17^ as major compounds, while from different Himalayan locations major compound reported was Z-β-ocimene followed by dihydrotagetone^12^. Keeping earlier studies as the basis for the present research, we hypothesized that seasonal variability in the Himalayan region could be an important feature to study the disparity in EO content and composition.

Spoilage of food is a major neglected issue caused by the production of microbial toxins^18^. The resistance of microorganisms has increased the use of synthetic chemicals, and it is, therefore, important to look for alternative agents like medicinal and aromatic plants to control microorganisms^19^. Thus, the antibacterial aspect of EO is an area with rising concern in last few decades, with efficiency even against multidrug-resistant strains ^20^. However, bioactive volatiles reported efficient antimicrobial activities^21^ and antimicrobial potential of the EOs of aromatic crops viz., Frankincense, myrtle, thyme, lemon, oregano, and lavender have been studied against pathogenic bacteria^22^. Besides antimicrobial activity, cytotoxic effects of some EOs viz., lemon verbena (*Lippia citriodora* Palau. Kunth)^23^, thyme (*Thymus vulgaris* L.)^24^ and rosemary (*R. officinalis*)^25^ have been reported earlier against some tumor cell lines.

The content and composition of EO often fluctuate among harvesting seasons and accessions^26^ and are additionally associated with genetic makeup, phenological phases (viz., reproductive or vegetative), altitude, soil, and climatic conditions^27^. To date, there has been inadequate information on the EO content from the Himalayan region on rosemary accessions harvested in different seasons and the dissimilarity that may exist in the compositions and biological properties. The main objective of the present work was to identify the accession and appropriate season for harvest with better quality EO which can put forward opportunities for superior cultivars selection for intended breeding endeavors. Additionally, to define a suitable season and accession for the growers in the region, it is necessary to analyze the variability in EO content, composition, and biological activity.

## Results

### Growth and biomass accumulation

Plant biometric observations viz*.,* plant height, number of branches, plant spread (E-W and N-S), fresh plant biomass (g plant^-1^) were significantly affected by harvesting seasons and accessions (Table 1). Significantly higher plant height and the number of branches in the summer season may be due to the rise in temperature and longer photoperiod (Table 1). Plant spread in both directions, i.e. NS (17.14% and 8.57%) and EW (16.21% and 10.81%) was higher in rainy season as compared with the summer and autumn seasons, respectively. Fresh biomass plant^-1^ was significantly higher (140.8 g) in the rainy season, followed by autumn (137.5 g) and summer (130.0 g) season. Rosemary accessions were not significantly different from each other in parameters viz., plant height, plant spread (E-W), and fresh biomass plant^-1^. The number of secondary branches was 11.5% higher in IHBT/RMAc-1 compared with IHBT/RMAc-2, while plant spread (N-S) was 10.0% higher in IHBT/RMAc-2 compared with IHBT/RMAc-1.

**Table 1:** Growth and biomass observations of *R. officinalis* accessions in different seasons of harvest and interaction of different seasons and accessions.

Treatment	Plant height (cm)	Number of secondary branches plant^-1^	Plant spread (cm)	
			N-S	E-W
**Harvesting seasons**				
Rainy	62.6^b^	26.7^b^	34.7^a^	37.0^a^
Autumn	61.3^bc^	26.2^b^	32.0^b^	33.3^b^
Summer	65.0^a^	31.7^a^	29.8^c^	31.8^c^
SE ( ±)	0.5	0.6	0.5	0.5
LSD (*P* = *0.05*)	1.7	2.0	1.8	1.7
**Accessions**				
IHBT/RM/Ac-1	63.0	29.1^a^	30.4^b^	33.1
IHBT/RM/Ac-2	62.3	26.0^b^	33.2^a^	34.3
SE ( ±)	0.4	0.5	0.4	0.4
LSD (*P* = *0.05*)	NS	1.6	1.5	NS

Interaction effect on plant biomass (g plant^-1^)				
Accessions	Rainy	Autumn	Summer	Mean (Accessions)
IHBT/RMAc-1	144.0^a^	131.0^de^	132.0^d^	135.7
IHBT/RMAc-2	137.7^c^	144.0^ab^	128.0^e^	136.6
Mean (Seasons)	140.8^A^	137.5^B^	130.0^C^	

		SE ( ±)	LSD (*P* = *0.05*)	
	Seasons	0.6	1.8	
	Accessions	0.5	NS	
	Interaction (Season × Accession)	0.8	3.0	

Means with the same superscripted letter in the same column did not differ significantly (*P* = 0.05); Interaction effect: similar superscripted letter indicate non significant differences and means without superscript letters do not differ statistically.

### Essential oil content and composition

**Figure 1:**
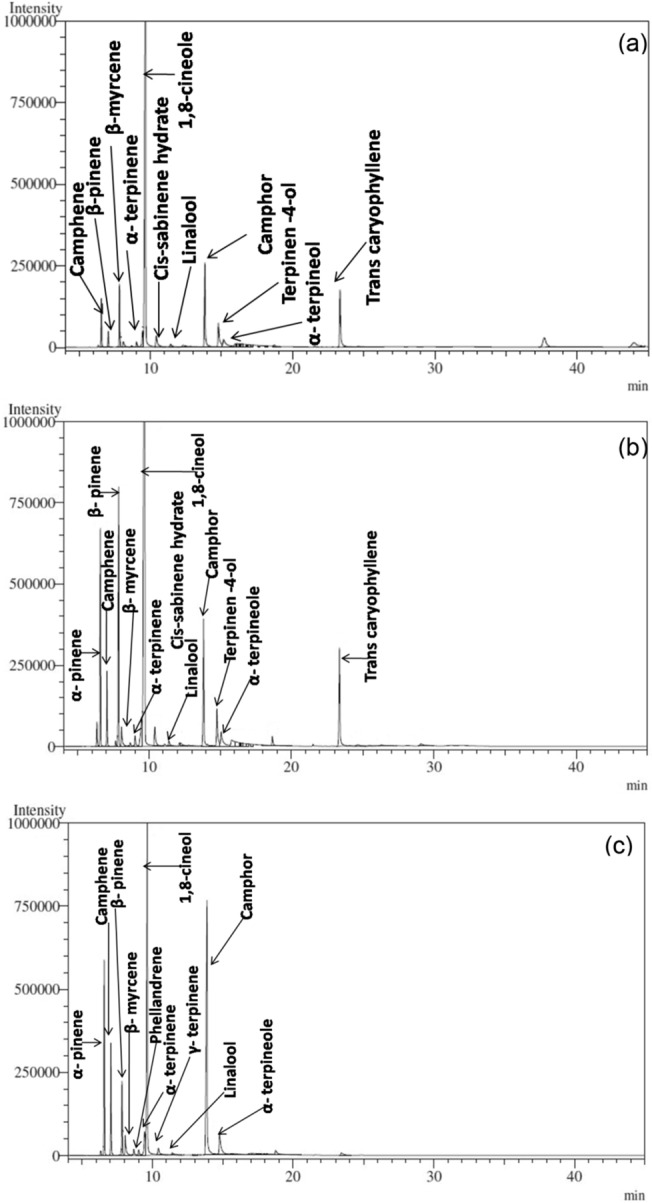
MS chromatograms of *R. officinalis* accession IHBT/RM/Ac-1 in (**a**) rainy season, (**b**) autumn season, and (**c**) summer season.

**Figure 2:**
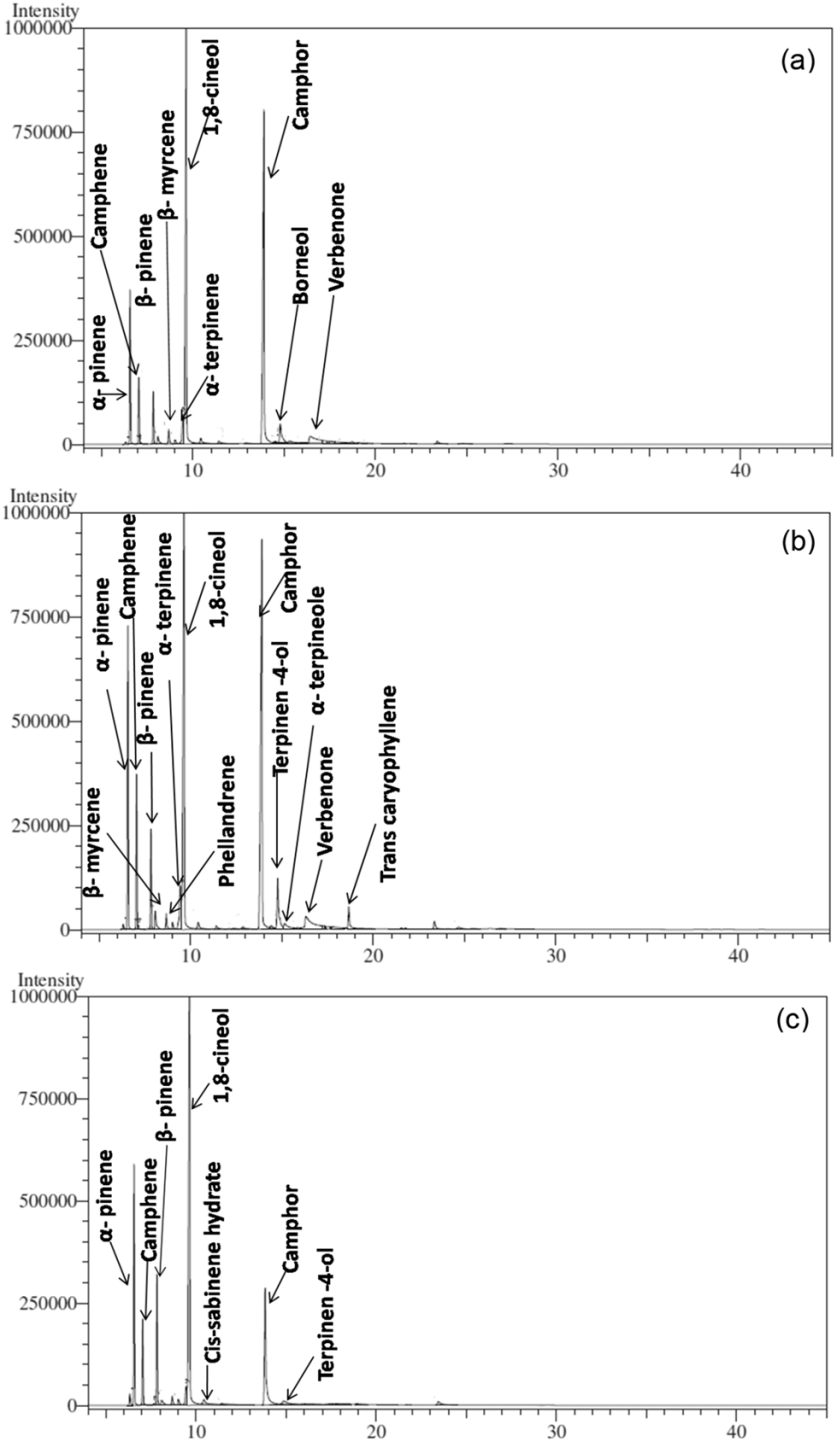
MS chromatograms of *R. officinalis* accession IHBT/RM/Ac-2 in (**a**) rainy season, (**b**) autumn season, and (**c**) summer season.

EO content extracted from aerial parts of *R. officinalis* plant was significantly affected by harvesting seasons and accessions (Table 2). The obtained EO was colourless and showed dominant camphorous and eucalyptus odor through visual and sniff test assessment, respectively. Significantly higher EO content was recorded during the autumn season (0.87%) as compared with summer (0.68%) and the rainy season (0.48%). Among accessions, IHBT/RMAc-1 recorded significantly higher (0.77%) content of EO in comparison to IHBT/RMAc-2 (0.59%) accession. The analysis of rosemary EO led to the detection and identification of fourteen chemical constituents which accounted for about 90.42 to 93.00% of the total EO area. The identified constituents are summarized in Table 2. The major compounds (area > 3%) were 1,8-cineole, camphor, α-pinene, camphene, β-pinene, and terpinen-4-ol with some minor compounds (area < 3%) viz., β-myrcene, α-terpinene, cis-sabinene hydrate, linalool, borneol, α-phellandrene, α-terpineol, and β-caryophyllene among harvesting seasons and accessions. Concerning the major six compounds in EO among three development harvesting stages, we found α-pinene, camphene, and α-terpinene showed the same trend of occurrence. The EO GC–MS chromatograms of accessions IHBT/RM/Ac-1 (Fig. 1) and IHBT/RM/Ac-2 (Fig. 2) in the rainy, autumn, and summer season were also illustrated.

**Table 2:** Essential oil content, constituents and grouped chemical component classes of *R. officinalis* over different harvesting seasons and accessions.

			Treatments								
			Harvesting seasons					Accessions			
			Rainy	Autumn	Summer	SE ( ±)	LSD (*P* = *0.05*)	IHBT/RMAc-1	IHBT/RMAc-2	SE ( ±)	LSD (*P* = *0.05*)
EO content (%)			0.48^c^	0.87^a^	0.68^b^	0.02		0.77^A^	0.59^B^	0.01	
EO constituents (%)	Litt. RI	Expt. RI (GCMS)									
α- pinene	932	935	5.47^c^	9.82^b^	12.8^a^	0.40	1.26	7.76^B^	10.9^A^	0.32	1.03
Camphene	946	950	3.50^c^	5.17^ab^	5.42^a^	0.14	0.43	5.24^A^	4.14^B^	0.11	0.35
β-pinene	974	975	7.60^a^	3.70^c^	5.88^b^	0.49	1.56	3.18^B^	8.28^A^	0.40	1.27
β –myrcene	988	984	2.77^a^	1.55^b^	1.18^c^	0.04	0.11	2.57^A^	1.10^B^	0.03	0.09
α-phellandrene	1008	1006	0.00	0.90^a^	0.88^b^	0.02	0.06	0.50^B^	0.69^A^	0.01	0.05
α-Terpinene	1014	1018	0.80^c^	1.97^ab^	1.98^a^	0.09	0.29	1.66	1.58	0.07	NS
1,8-cineole	1026	1027	51.79^a^	32.50^c^	37.35^b^	0.60	1.91	38.70^B^	42.20^A^	0.49	1.56
Cis-sabinene hydrate	1065	1063	1.42^a^	0.38^c^	0.75^b^	0.06	0.20	0.73^B^	0.97^A^	0.05	0.16
Linalool	1095	1099	0.00	0.50^a^	0.00	0.01	0.04	0.40^A^	0.17^B^	0.01	0.03
Camphor	1141	1146	7.05^c^	31.50^a^	22.00^b^	0.71	2.27	22.17^A^	18.20^B^	0.58	1.85
Borneol	1165	1172	0.00	1.45^a^	0.00	0.04	0.14	0.00^B^	0.97^A^	0.04	0.12
Terpinen-4-ol	1174	1174	2.62^a^	1.78^c^	2.48^ab^	0.16	0.50	3.64^A^	0.94^B^	0.13	0.40
α-terpineol	1186	1181	1.10^ab^	1.78^a^	0.58^c^	0.11	0.36	2.31^A^	0.00	0.09	0.30
β-caryophyllene	1408	1414	6.30^a^	0.00	1.00^b^	0.14	0.50	2.74^A^	2.13^B^	0.12	0.40
**Component classes (%)**											
Monoterpenes			21.50^bc^	23.50^b^	29.00^a^	0.95		21.60^B^	27.70^A^	0.78	
Oxygenated Monoterpenes			62.62^b^	69.50^a^	62.30^bc^	0.87		67.30^A^	62.50^B^	0.71	
Sesquiterpenes			6.30^a^	0.00	1.00^b^	0.14		2.70^A^	2.17^B^	0.12	
Total area (%)			90.42	93.00	92.30			91.50	92.37		

Means with the same letter in the same row did not differ significantly (*P* = 0.05).

Significantly higher 1,8-cineole, β-pinene, cis-sabinene hydrate, and terpinen-4-ol were recorded in the rainy season as compared with other seasons but terpinen-4-ol remained at par with the summer season. The content of β-caryophyllene was significantly higher in the rainy season as compared with summer while not recorded during the autumn season although camphor was highest in autumn as compared with summer and the rainy seasons. Similarly, α-phellandrene was also higher in the autumn season but remained at par with summer and was absent during the rainy season. Significantly higher α-pinene was recorded in the summer season compared with autumn and rainy season while camphene and α-terpinene were also higher during the summer compared to rainy season but remained at par with autumn season. Significantly higher 1,8-cineole, α-pinene, β-pinene, α-phellandrene, and borneol were recorded in IHBT/RMAc-2 as compared with IHBT/RMAc-1. At the same time, camphor, camphene, β-myrcene, α-terpinene, linalool, terpinen-4-ol, α-terpineol, and β-caryophyllene were significantly higher in IHBT/RMAc-1 as compared with IHBT/RMAc-2 accession.

### Interaction effect

The interaction effect of harvesting seasons and accessions was significant on plant biomass (Table 1) and few EO constituents (Table 3). The interaction between season and accession showed that the highest plant biomass was observed in IHBT/RMAc-1 during the rainy season (144.0 g plant^-1^) followed by IHBT/RMAc-2 accession during the autumn season compared to other treatments (Table 1). Similarly, a significant interaction between harvesting season and accessions was observed for α-pinene, 1,8-cineole, and camphor (Table 3). Accession IHBT/RMAc-2 harvested during the summer season recorded significantly higher α-pinene content followed by IHBT/RMAc-1 harvested during the autumn season compared to other treatments. Similarly, the interaction between season and accessions showed higher 1,8-cineole content in IHBT/RMAc-1 during the rainy season followed by IHBT/RMAc-2 in the same season compared to other treatments. The interaction effect of harvesting season and accession showed higher camphor content in IHBT/RMAc-2 harvested during the autumn season followed by IHBT/RMAc-1 in the same season.

**Table 3:** Effect of season and accession and their interaction on major essential oil constituents of *R. officinalis*.

α- pinene	Harvesting seasons			
Accessions	Rainy	Autumn	Summer	Mean (Accessions)
IHBT/RMAc-1	0.0f.	11.7^b^	11.6^bc^	7.8^B^
IHBT/RMAc-2	10.9^bcd^	8.0^e^	14.0^a^	11.0^A^
Mean (Seasons)	5.5^C^	9.8^B^	12.8^A^	

	SE ( ±)	LSD (*P* = *0.05*)		
Seasons	0.4	1.3		
Accessions	0.3	1.0		
Interaction (Season × Accession)	0.6	1.8		

1,8-cineole	Harvesting seasons			
Accessions	Rainy	Autumn	Summer	Mean (Accessions)
IHBT/RMAc-1	55.4^a^	32.0^de^	28.7f.	38.7^B^
IHBT/RMAc-2	48.1^b^	33.0^d^	45.4^bc^	42.2^A^
Mean (Seasons)	51.8^A^	32.5^C^	37.1^B^	

	SE ( ±)	LSD (*P* = *0.05*)		
Seasons	0.6	1.9		
Accessions	0.5	1.6		
Interaction (Season × Accession)	0.8	2.7		

Camphor	Harvesting seasons			
Accessions	Rainy	Autumn	Summer	Mean (Accessions)
IHBT/RMAc-1	8.7^e^	29.7^b^	28.2^bc^	22.2^A^
IHBT/RMAc-2	5.4f.	33.3^a^	15.8^d^	18.2^B^
Mean (Seasons)	7.05^C^	31.5^A^	22^B^	

	SE ( ±)	LSD (*P* = *0.05*)		
Seasons	0.7	2.3		
Accessions	0.6	1.9		
Interaction (Season × Accession)	1.0	3.2		

Means with the same lower case superscripted letter indicate the interaction effect and means with similar superscripted letter did not differ significantly (*P* = 0.05); values indicated with upper case superscripted letter are the means and different upper case superscripted letters indicate significant difference (*P* = 0.05).

### Grouped components

Grouped components were differentiated by a major contribution of oxygenated monoterpenes (62.30–69.50%) followed by monoterpenes (21.50–29.0%) and sesquiterpenes hydrocarbons (0.0–6.3%) (Table 2). EO of *R. officinalis* during the summer season recorded 21.4% and 31.8% higher monoterpenes as compared with autumn and rainy seasons, while autumn season recorded 10.7% and 11.5% higher oxygenated monoterpenes as compared with rainy and summer seasons, respectively. Significantly higher sesquiterpenes were recorded in the rainy as compared with summer while absent in the autumn season. Oxygenated monoterpenes and sesquiterpenes were 7.52% and 28.6% higher in IHBT/RMAc-1 as compared with IHBT/RMAc-2 while monoterpenes were 25.02% higher in IHBT/RMAc-2 as compared with IHBT/RMAc-1.

### Principal component analysis (PCA)

**Figure 3:**
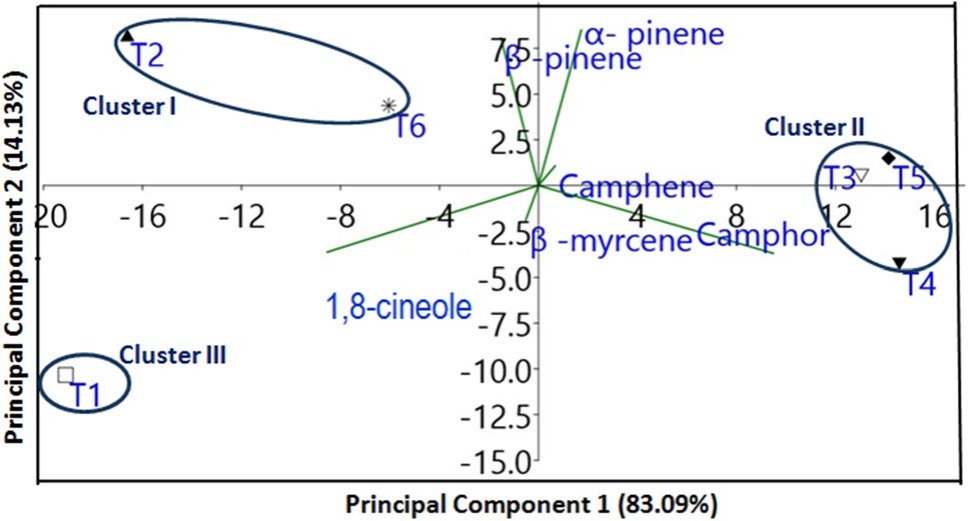
Principal component 1 and Principal component 2 jointly explained 97.22% of the total variation for T1, T2, T3, T4, T5 and T6 where T1: Rainy season IHBT/RMAc-1; T2: Rainy season IHBT/RMAc-2; T3: Autumn season IHBT/RMAc-1; T4: Autumn season IHBT/RMAc-2; T5: Summer season IHBT/RMAc-1; T6: Summer season IHBT/RMAc-2.

The identified components among harvesting seasons and accessions of rosemary EO were subjected to principal component analysis (PCA) sequentially to analyze variability in the various treatments. The ordination analysis results with the first two principal components were presented through a bi-plot (Fig. 3); the acute and obtuse angles of variable vectors denote the level of positive and negative associations among variables, respectively. PC-1 and PC-2 come up with 83.09% and 14.13% of the variance, respectively, which mutually accounted for 97.39% of the total variance (Fig. 3). Seasonal variation among rosemary accessions illustrated three clusters in a scatter plot. The data points of the variables with similarities were grouped in the same clusters of PCA. Cluster I included intermediate concentrations of major compounds viz., 1,8-cineole, and α-pinene in treatments T2 and T6 while cluster II included lowest concentrations of major compounds 1,8-cineole and intermediate concentration of camphor and α-pinene in T3, T4, and T5. Cluster III was independent and included the highest concentrations of major compounds 1,8-cineole, lower concentration of camphor, and absence of α-pinene.

**Figure 4:**
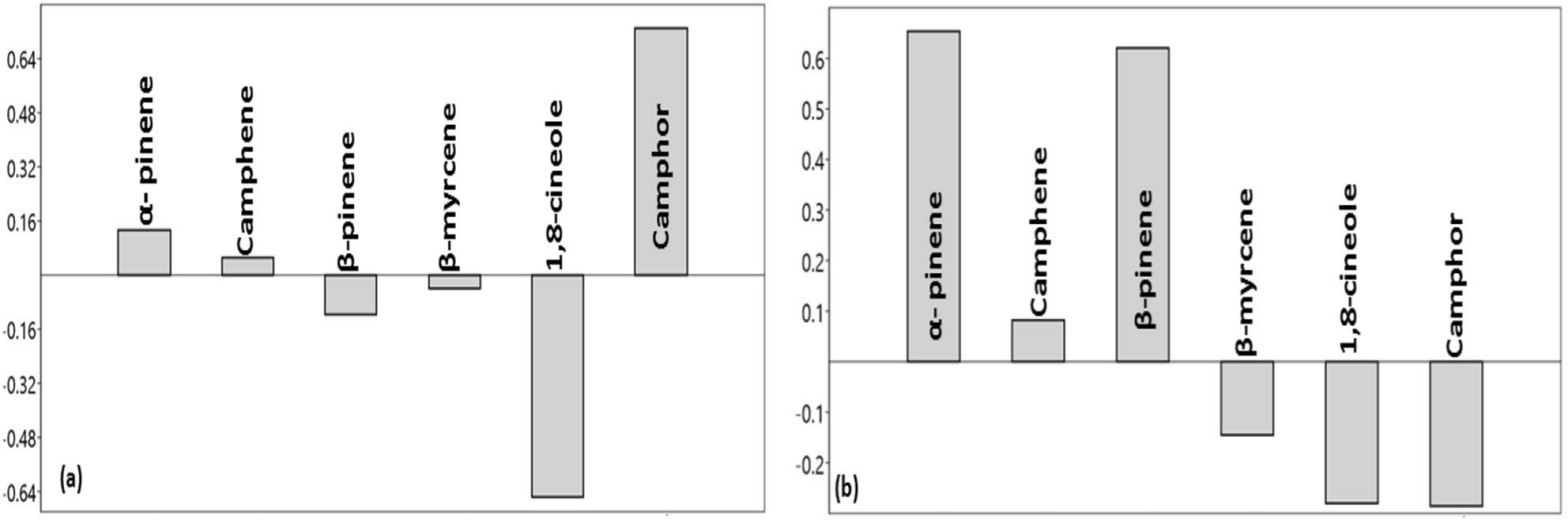
Loading plot of principal component analysis of different seasons and accessions (**a**) loading plot PC-1, (**b**) loading plot PC-2.

PC-1 of seasonal variability among accessions demonstrated that α-pinene, camphene, and camphor had positive loading, whereas β-pinene, β-myrcene, and 1,8-cineole were negatively loaded (Fig. 4a). PC-2 was chiefly distributed with positive loading of α-pinene, camphene, and β-pinene while β-myrcene, 1,8-cineole, and camphor showed negative loading (Fig. 4b). However, the percentage of main constituents (1,8-cineole) decreased from the rainy to the summer season (Table 4) with the cluster variability of major EO constituents (%) in EO in different seasons of harvesting.

**Table 4:** Clusters variability in major essential oil constituents (%) of *R. officinalis* with the variation in seasons and accessions.

EO Components	Cluster I	Cluster II	Cluster III
α- pinene	10.9–14.0	8.0–11.7	0.0
Camphene	3.8–5.1	3.5–6.8	3.2
β -pinene	7.9–14.0	2.9–4.5	1.2
β -myrcene	1.1–1.2	1.0–2.1	4.3
1,8-cineole	45.4–48.1	2.8.7–33.0	55.4
Camphor	5.4–15.8	28.2–33.3	8.7

### Antibacterial and cytotoxic activity

The antibacterial evaluation of rosemary EOs illustrated significant inhibitory activity against Gram-positive (both) bacterial strains and one Gram-negative strain *S. typhi* MTCC 733, while *K. pneumoniae* MTCC 109 was found resistant (Table 5). Strains *S. aureus* MTCC 96 and *S. typhi* MTCC 733 were inhibited by EOs of all the seasons and accessions. All the treatment combinations displayed an inhibitory effect against all the tested strains except *K. pneumoniae* MTCC 109. The EOs of the rainy season with accession IHBT/RM/Ac-1 exhibited significantly higher antibacterial activity for *S. aureus* MTCC 96, as compared with the summer season along with accession IHBT/RM/Ac-2. Conversely, the EOs extracted during the summer season showed a significantly higher inhibitory effect against *S. typhi* MTCC 733 than that of the rainy season. Regardless of the inhibitory effect of the EOs, only one strain, i.e. *S. aureus* MTCC 96 was marked as sensitive (≥ 8 mm) to the EO of the rainy season and accession IHBT/RM/Ac-1, elucidating a maximum zone of inhibition of 14 mm. The minimum inhibitory concentration of rosemary EO was evaluated using the broth microdilution assay against the bacterial strain *S. aureus* MTCC 96 as it showed sensitivity in well diffusion assay. The MIC was observed at 4% (v/v). MTT assay was performed to examine the effect of different treatments of rosemary EO on cellular viability of A549 and CAL 27 cancerous cell lines. Results of MTT assay showed that rosemary EO did not show any cytotoxic effects against both the cell lines, i.e. A549 and CAL 27 at varying concentrations (10, 25, 50, 100, and 200 µg mL^-1^) at 24, 48, and 72 h of incubation.

**Table 5:** Antibacterial activity of the *R. officinalis* essential oils on Gram-positive and Gram-negative bacterial strains.

Treatments	GRAM-POSITIVE		GRAM-NEGATIVE	
	*Staphylococcus aureus* MTCC96	*Micrococcus luteus* MTCC2470	*Salmonella typhi* MTCC733	*Klebsiella pneumoniae* MTCC109
T 1	14.00 ± 1.7	–	5.33 ± 1.2	–
T 2	7.33 ± 0.5	–	5.00 ± 0.0	–
T 3	7.33 ± 0.5	5.00 ± 1.0	4.66 ± 0.5	–
T 4	5.33 ± 0.5	5.00 ± 0.0	8.66 ± 0.5	–
T 5	5.33 ± 0.5	5.33 ± 1.15	7.00 ± 0.0	–
T 6	5.66 ± 0.5	–	7.00 ± 0.0	–

Ampicillin (10 µg disc^-1^) and streptomycin (10 µg disc^-1^) were used as positive controls against Gram-positive and Gram-negative bacteria, respectively; Values are means ± Standard Deviation (SD) of triplicate readings expressed in mm including 6 mm of well diameter.

T1: Rainy season IHBT/RMAc-1; T2: Rainy season IHBT/RMAc-2; T3: Autumn season IHBT/RMAc-1; T4: Autumn season IHBT/RMAc-2; T5: Summer season IHBT/RMAc-1; T6: Summer season IHBT/RMAc-2.

## Discussion

**Figure 5:**
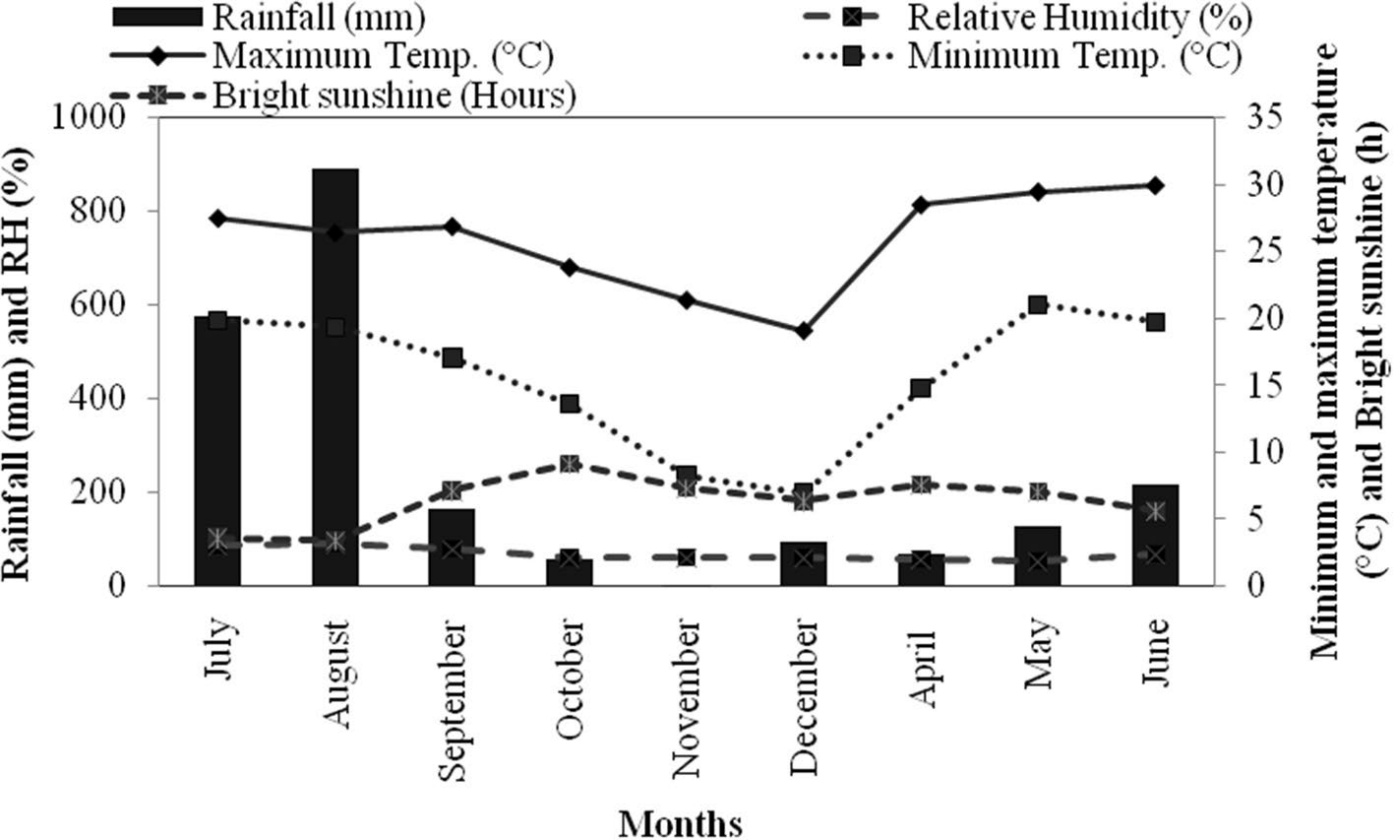
Mean monthly (seasonal) meteorological data during crop growth season (2018–2019) of rosemary at Palampur, Himachal Pradesh, India.

Plant height and the number of secondary branches were higher in the summer season than autumn and rainy seasons, which may be due to the rise in temperature and longer photoperiod in the respective season (Fig. 5). Plant spread in both directions, i.e. NS and EW were higher in the rainy season as compared with summer and autumn seasons. The recorded biomass of rosemary was highest in rainy season and lowest in the summer season which may be attributed to the low rainfall and high temperature in the summer season months, which ultimately resulted in decreased biomass. The consequence of a minimum temperature (19 °C) in the rainy season was expected to induce a significant seasonal plant fresh biomass increase and decreased EO content. A reverse tendency was observed during the autumn season when minimum temperature (9 °C) brought a subsequent decrease in plant fresh biomass which ultimately resulted in high EO content. The flowering stage occurred during the autumn and summer months but was accompanied by less rainfall and lower temperatures which accounted for lower biomass. Analogous results have been reported in palmarosa with high plant biomass during the rainy season in plentiful soil moisture conditions^28^ and biomass accumulation varies with factors viz., environment, and crop phenology^29^. The plant biomass disparity usually can be correlated to temperature, harvesting stage, rainfall, and also influenced by varied climatic conditions in peppermint (*Mentha piperita* L.)^30^.

The lower EO content in the summer season was because of comparatively higher temperatures (26–30 °C) in comparison to other harvesting seasons, which probably resulted in the loss of volatile compounds from aerial parts of the plant. During the autumn and summer seasons, the phenological stage of flowering in rosemary plants contributed to total EO content in addition to leaves and twigs in contrast to the rainy season. The flowering season at the research work site starts during the end of the summer season and continues up to the beginning of the autumn season, which corresponded to the highest EO content in the autumn and summer season, while the lowest during the rainy season was related to the vegetative stage of the plant. Developmental and flowering phases of the aromatic plant affect the EO content and availability; analogous findings with higher EO content were reported in *Nectandra* spp.^31^ and *Achillea fragrantissima* (Forssk.) Sch.Bip.^32^ during the autumn and summer seasons, respectively. The EO content disparity in accessions was due to inherent features such as genetic makeup and origin of the plant^15^. The present results showed that the studied rosemary accessions in the western Himalayan region had higher EO content than earlier reports^33^. On the contrary, the EO content in studied Iranian rosemary accessions was higher than present findings and ranged from 0.53 to 2.60%^15^. Similar findings with higher accumulation of EO were observed in mint accessions at similar altitudinal conditions and EO production depends on adaptation mechanism through oil glandular trichomes density variation and associated with the growing conditions^34^. The EO content in the present finding among seasons ranged from 0.48% to 0.87%, which is similar to the earlier findings on seasonal variability in rosemary^5,16^.

Among all treatments, 1,8-cineole and camphor contributed to about 50% of the EO constituent composition. According to previous findings, camphor and verbenone were reported as major constituents^35^ of rosemary EO. Similar to present findings rosemary accessions reported 1,8-cineole as major EO constituent followed by camphor^15^. The seasonal variability in varied locations recorded camphor^16^ and α-pinene^5^ as chief EO constituents followed by 1,8-cineole in rosemary. Additionally, EO extracted of rosemary genotypes in different seasons under different extraction methods observed 1,8-cineole, camphor, verbenone, and α-pinene as leading constituents, thus reported four different chemotypes^36^. These variations can be attributed to many factors, viz., genetic dissimilarity within accessions, climate variability, and origin of plants in addition to drying, storage, and extraction processes^36^. The quantitative differences detected in 1,8-cineole, and camphor content in the present study are mainly dependent on seasonal harvesting. The chemical composition of the plants gets influenced by the accumulation of a few compounds at a particular period of the season in response to environmental conditions and seasons^37–39^. The concentration of major EO compound (1,8-cineole) was recorded to be highest among seasons, while other EO constituents amount differed randomly during the plant life cycle. *R. officinalis* EOs are chiefly of four chemotypes: α-pinene chemotype (from Spain, Iran, Italy, Romania, and France), 1,8-cineole chemotype (from Algeria, Morocco, and Austria), camphor chemotype (from India and Cuba), and myrcene chemotype (from Portugal and Argentina)^40^. The major component of EO in our region was 1,8-cineole (among all treatments) followed by camphor and is, therefore, 1,8-cineole chemotype of rosemary while some other studies reported α-pinene rich^41^ and camphor rich chemotype^42^ in tropical climatic conditions of Brazil and subtropical climatic conditions Northern India, respectively. In contrast, to present findings, the composition of rosemary EO studied in coastal Mediterranean environment recorded α-pinene as the main constituent (up to 75%) followed by eucalyptol among all sampling seasons^43^. Similar to the present findings, the presence of high monoterpenes compounds as compared to other components classes was also reported in rosemary, tea tree (*Melaleuca alternifolia* Maiden & Betche Cheel), and pine (*Pinus pinaster* Aiton)^44^. In contrast, seasonal variations in EO of rosemary grown in the warm hot Mediterranean climate of Sardinia recorded six EO classes of hydrocarbons viz., monoterpenes, alcohols, aldehydes, ketones, esters, and sesquiterpene^5^. Additionally, similar to present findings seasonal and genotypic variability in the volatile profile of rosemary was primarily dominated by mono- and sesquiterpenes which contributed to about 85 to 90% of the total EO volatiles, and rest is followed by alcohols, esters, and aldehydes^36,45^. Additionally, monoterpenes are reported to be synthesized and stored in secretory organs and emission of monoterpenes seems to be a temperature-driven diffusion process corresponding to this fact significant emissions of monoterpenes were reported during the summer season^46^. Moreover, the terpene storage is usually reported to be maximum in the autumn–winter period, but emissions might differ among species, but usually found maximum during the spring–summer period^47^. The rosemary EO is proficient in curbing the growth of tested Gram-positive bacteria as inferred from the current study. The antibacterial activities of rosemary EOs against the Gram-positive *S. aureus* are in harmony with previous reports where rosemary EOs showed antimicrobial activity against *Staphylococcus* spp*.*, *Bacillus* spp*.*, *Escherichia* spp*.,* and *Candida* spp. and *Pseudomonas aeruginosa*^48–51^. EO was ineffective against *K. pneumoniae* which might be because the antibacterial action of EO depends on its composition, functional groups and varies for each microbial strain. The higher vulnerability of Gram-positive bacteria as compared with the Gram-negative bacteria to EOs is an established fact^52^. The Gram-positive cell membrane contains lipoteichoic acid that may favor easy accessibility of hydrophobic EOs, whereas the rigid and complex lipopolysaccharide of Gram-negative membrane restricts the entry of hydrophobic compounds^52^. The hydrophobic character of the EO helps it to partition with cell and mitochondrial lipids that cause membrane permeability. This leads to leakage of essential molecules, eventually causing cell death^53^. In the current study, EOs obtained during the rainy season has better antibacterial activity against the Gram-positive *S. aureus* as compared with EOs of other seasons. A previous study on the effects of seasonal variation on the composition and biological activities of rosemary EOs reported that the EOs harvested during the summer season exhibited superior antibacterial activity against *S. aureus*^26^. The better antibacterial activity exhibited by EOs of the rainy season and accession IHBT/RM/Ac-1 may be attributed to the occurrence of active components 1,8-cineole, and camphor in higher levels^26^.

The EO of rosemary in the present study did not show any cytotoxic effects against both the cell lines i.e. A549 and CAL 27, in contrast, previous studies on rosemary EO showed toxic effects on epithelial cancer cells and lethality to mice at 5.50 g kg^-1^ dosage^54,55^. This is a well-proven fact that many therapeutic effects of the EOs have been attributed to amounts of both major and minor constituents of EO^20^. Our study recorded no cytotoxic activity of EO in both cell lines; this was due to the presence of 1,8-cineole (as major compound) which accounted for low toxicity on tumor cell lines^56^. Comparable findings have been accounted for with no cytotoxicity of rosemary EO (rich in 1,8-cineole) on cancerous cell lines^57^ while cytotoxic potential on human-derived macrophage THP-1 cells with rosemary EO (rich in α-pinene) was observed^25^. All the unpredictability in the performance of EO can be attributed to the compositional variability of EO in the region.

## Conclusions

In the present study, the EO content was highest during the autumn season and revealed the presence of oxygenated monoterpenes, monoterpenes, and a smaller amount of sesquiterpenes. Major compound viz., 1,8-cineole, was significantly higher in the rainy season, although crop can be harvested accordingly in different seasons as per the need of compounds by growers and industry. The present findings did not highlight the cytotoxic potential of rosemary EO against the cell lines but have been reported to display antimicrobial activity. The results specified that *S. aureus* MTCC 96, in particular, was the most sensitive bacteria to rosemary EO. So, we could infer that the rosemary EO can find application as a potential natural antimicrobial agent against *S. aureus* in the food and pharmaceutical industries. We can conclude that to obtain a higher oil yield, rosemary should be harvested in the autumn season, and both accessions have the potential to contribute to breeding programs which can lead to the development of desired cultivars in harmony with industrial requirements from the western Himalayan region.

## Methods

### Experimental site

An experiment was executed during 2018–2019 in the experimental area of CSIR-IHBT (Council of Scientific and Industrial Research- Institute of Himalayan Bioresource Technology), Palampur, Himachal Pradesh (HP), India; situated at 1325 m above mean sea level (amsl) altitude (32°11ʹ39ʹʹN latitude and 76°56ʹ51ʹʹE longitude). The climate of this site is subtropical and soil can be described as mollisol, with neutral pH. Weather parameters viz*.,* minimum and maximum temperature (°C), relative humidity (RH%), and average bright sunshine (BSS) hours during the seasonal crop harvesting were obtained from agro-meteorological advisory “Crop weather outlook”^58^ and depicted in Fig. 5. Maximum (30 °C) and minimum (4 °C) temperatures were recorded in June and January, respectively. Mean relative humidity was maximum (90%) in August and minimum (53%) in May months. Total rainfall received in Palampur region during rosemary growth seasons was 228 cm, with maximum in August and lowest in November month while, average daily BSS received was 7 h, during experiment duration.

### Experimental details

The academic permission for the cultivation of rosemary accessions was obtained from the Director, CSIR-IHBT, Palampur, Himachal Pradesh, India, and the study complies with all relevant guidelines. The present research consisted of factorial experiment (two factors) with six treatment combinations, i.e. three harvesting seasons viz., rainy, autumn, and summer seasons and two accessions viz*.,* IHBT/RMAc-1 and IHBT/RMAc-2 with treatment combinations of T1: Rainy season IHBT/RMAc-1; T2: Rainy season IHBT/RMAc-2; T3: Autumn season IHBT/RMAc-1; T4: Autumn season IHBT/RMAc-2; T5: Summer season IHBT/RMAc-1; T6: Summer season IHBT/RMAc-2. The accession IHBT/RMAc-1 has bluish coloured flowers and dark green leaves while IHBT/RMAc-2 has white coloured flowers and light green leaves. Both the accessions were not indigenous and were planted in pots (pot size 35 cm × 35 cm × 23 cm) containing sand, soil, and decomposed farmyard manure (FYM) in the ratio of 1:1:1. Rosemary plants were planted in nursery beds during December 2015; then transplanted in pots after a year and at the time of harvesting the plants were three years old.

### Growth yield and EO extraction

Various biometric observations viz., plant height (cm), plant spread (cm) in both directions i.e. north–south (N–S) and east–west (E–W), and the number of branches per plant were recorded during the rainy (flowering stage), summer (flowering stage), and autumn (vegetative stage) season (Table 1). The plant harvest was initiated after 605, 764, and 830 days after transplanting during the rainy, autumn, and summer season, respectively. Plant biomass (g plant^−1^; five plants from each treatment) was recorded at the time of harvest on per plant basis in rainy (flowering stage), autumn (vegetative stage), and summer (flowering stage) seasons, which includes the aerial part of the plant. From each treatment, 500 g fresh aerial parts viz., leaves, twigs, and flowers were collected and hydrodistilled (4 h) in triplicates in the Clevenger-type apparatus^59^. The EO content was detailed based on v/w (%) criteria and dried with anhydrous sodium sulfate (Na_2_SO_4_). The EO was collected in a dark glass container as well as stored at 4 °C before analysis.

### Gas chromatography, gas chromatography/mass spectrometry and Identification of components

GC and GC/MS analysis were performed through flame ionization detector (FID) on Shimadzu GC 2010 gas chromatograph and QP2010 (Shimadzu Corp., Tokyo, Japan) fitted with an AOC 5000 auto-injector. The auto-injector comprises of 30 m long ZB-5 MS capillary column with 0.25 mm i.d. and 0.25 μm thick film (SGE International, Ringwood, Australia). Ten μL of EO was dissolved in 2 mL of dichloromethane with 2μL auto-injection volume in split mode. Carrier gas (nitrogen) with 1.05 mL min^-1^ flow rate; oven temperature of 70 °C for 3 min and afterwards to 220 °C for 5 min at the rate of 4 °C min^-1^ and injector and detector temperature was programmed at 220 °C and 250 °C, respectively. The temperature programming, injection volume, and carrier gas conditions utilized for performing GC and GC/MS were as per the procedure mentioned in German chamomile (*Matricaria chamomilla* L.)^60^. For identification and recognition of compounds, a series of hydrocarbons was utilized for retention index (RI) estimation (Table 2). The EO components were identified through harmonizing the experimental RIs with the reported RIs in literature^41,61^. The components were also identified by matching the minimum mass spectral fragmentation pattern of the components with the NIST library^62^.

### Antibacterial activity: agar well-diffusion method

EO of *R. officinalis* was screened for antibacterial activities following a well diffusion method^63^. The evaluated bacterial strains were *Micrococcus* *luteus* MTCC 2470 and *Staphylococcus aureus* MTCC 96 (Gram-positive) and *Klebsiella pneumoniae* MTCC 109 and *Salmonella typhi* MTCC 733 (Gram-negative). The culturing and incubation conditions followed for antimicrobial activity evaluation of bacterial strains were same as previously detailed^12^. The positive controls taken against bacterial strains were Ampicillin at 10 µg disc^-1^ (Gram-positive) and streptomycin at 10 µg disc^-1^ (Gram-negative). The plates were allowed to disperse EOs in the agar medium as incubated for 2-3 h at 4 °C, then 12-24 h for 37 °C. The zones of inhibition were measured after performing the tests in triplicates. The categorization of EOs sensitivity was done as not sensitive, sensitive, and very sensitive with a diameter of ≤ 8 mm, 9-14 mm and ≥ 15 mm, respectively^64^.

### Determination of minimum inhibitory concentration (MIC)

The MIC of the EO against the bacterial strain showing sensitivity in agar well diffusion assay was determined by broth microdilution assay in a 96-well microtitre plate^65^. Overnight bacterial culture was regulated to 0.5 McFarland standards. The EO was diluted in MHB containing 0.5% (v/v) Tween 20 as emulsifier to obtain the concentration ranges from 8% (v/v) to 0.125% (v/v) in a microplate. To the prepared EO dilutions, 50µL of the prepared bacterial suspension was introduced in each well and after incubation of 12-24 h at 37 °C, 30µL of resazurin (0.015% w/v) was added to all wells. After further incubation for 2-4 h, a change in color from blue to pink was visually observed. The MIC is the lowest concentration with no visible color change. 100µL of broth medium in one column represented control for sterility check.

### Cell culture maintenance

A549 (human lung carcinoma) and CAL27 (squamous cell carcinoma) cell lines were acquired from ATCC (USA). The procured cells were preserved in DMEM (Dulbecco’s modified Eagles medium) with 10% FBS (fetal bovine serum), 1% antibiotic, and anti-mycotic at 37 °C with 5% CO_2_.

### Cellular viability assay

The proliferation rates of A549 and CAL27 cells were verified through colorimetric assay i.e. 3-(4, 5-dimethylthiazol-2-yl)-2, 5-diphenyl tetrazolium bromide (MTT) after treating them with different concentrations of EO. A549 and CAL 27 were seeded at a concentration of 10^4^ cells per well in a flat-bottom culture plate with 96-well and grown in a complete growth medium under standard culturing conditions. After overnight attachment of cells, they were treated with the presence or absence of diverse concentrations of volatile oil (10, 25, 50, 100, and 200 µg mL^-1^) then incubated up to 24, 48, and 72 h at 37 °C with 5% CO_2_. Subsequently, 5µL of MTT solution (5000 µg L^-1^ stock) was supplemented to wells then incubated up to 4 h at 37 °C. Viable cells were capable of reducing MTT via the action of mitochondrial dehydrogenases into water-insoluble blue-colored formazan crystals^66^. In the final step, 100μL of dimethyl sulfoxide (DMSO) was added to each one well after removing the solution trailed by 30 min incubation. To ensure the complete solubilization of formazan crystals plate was shaken for 2 min and measured absorbance at 570 nm with spectrophotometric microplate reader (Synergy, BioTek, United States).

### Statistical analysis

A completely randomized design (CRD) was utilized with three replications for analyzing data statistically. Fisher’s least significant difference (LSD) test was performed for least significant testing and regarded as statistically significant at *P* = 0.05. Multivariate principal component analysis (PCA software PAST3) was employed to assess the expression and the effect of harvest seasons and accessions on EO compounds.
